# Augmentation of arginase 1 expression by exposure to air pollution exacerbates the airways hyperresponsiveness in murine models of asthma

**DOI:** 10.1186/1465-9921-12-19

**Published:** 2011-02-03

**Authors:** Michelle L North, Hajera Amatullah, Nivedita Khanna, Bruce Urch, Hartmut Grasemann, Frances Silverman, Jeremy A Scott

**Affiliations:** 1 Institute of Medical Science, Faculty of Medicine, University of Toronto, Toronto, ON, Canada; 2 Divisions of Occupational and Respiratory Medicine, Department and Faculty of Medicine, University of Toronto, Toronto, ON, Canada; 3 Gage Occupational and Environmental Health Unit, University of Toronto and St. Michael's Hospital, Toronto, ON, Canada; 4 Keenan Research Centre in the Li Ka Shing Knowledge Institute of St. Michael's Hospital, Toronto, ON, Canada; 5 Division of Occupational and Environmental Health, Dalla Lana School of Public Health, Faculty of Medicine, University of Toronto, Toronto, ON, Canada; 6 Program in Physiology and Experimental Medicine, Research Institute, and Division of Respiratory Medicine, Department of Paediatrics, The Hospital for Sick Children, University of Toronto, Toronto, ON, Canada

## Abstract

**Background:**

Arginase overexpression contributes to airways hyperresponsiveness (AHR) in asthma. Arginase expression is further augmented in cigarette smoking asthmatics, suggesting that it may be upregulated by environmental pollution. Thus, we hypothesize that arginase contributes to the exacerbation of respiratory symptoms following exposure to air pollution, and that pharmacologic inhibition of arginase would abrogate the pollution-induced AHR.

**Methods:**

To investigate the role of arginase in the air pollution-induced exacerbation of airways responsiveness, we employed two murine models of allergic airways inflammation. Mice were sensitized to ovalbumin (OVA) and challenged with nebulized PBS (OVA/PBS) or OVA (OVA/OVA) for three consecutive days (sub-acute model) or 12 weeks (chronic model), which exhibit inflammatory cell influx and remodeling/AHR, respectively. Twenty-four hours after the final challenge, mice were exposed to concentrated ambient fine particles plus ozone (CAP+O_3_), or HEPA-filtered air (FA), for 4 hours. After the CAP+O_3 _exposures, mice underwent tracheal cannulation and were treated with an aerosolized arginase inhibitor (*S*-boronoethyl-L-cysteine; BEC) or vehicle, immediately before determination of respiratory function and methacholine-responsiveness using the flexiVent^®^. Lungs were then collected for comparison of arginase activity, protein expression, and immunohistochemical localization.

**Results:**

Compared to FA, arginase activity was significantly augmented in the lungs of CAP+O_3_-exposed OVA/OVA mice in both the sub-acute and chronic models. Western blotting and immunohistochemical staining revealed that the increased activity was due to arginase 1 expression in the area surrounding the airways in both models. Arginase inhibition significantly reduced the CAP+O_3_-induced increase in AHR in both models.

**Conclusions:**

This study demonstrates that arginase is upregulated following environmental exposures in murine models of asthma, and contributes to the pollution-induced exacerbation of airways responsiveness. Thus arginase may be a therapeutic target to protect susceptible populations against the adverse health effects of air pollution, such as fine particles and ozone, which are two of the major contributors to smog.

## Background

Epidemiological studies have described a relationship between ambient levels of air pollution, and respiratory admissions to hospitals [1,2]. It has become increasingly imperative to determine the biological effects of urban air pollutants, as they pose a serious risk to public health and continue to present an enormous and increasing health and economic burden [3,4]. Investigations of the health impact of air pollution using controlled human exposures have demonstrated acute cardiopulmonary effects in both healthy subjects and asthmatics [5-7]. Fine particulate matter, with an aerodynamic diameter of less than 2.5 μm, has been specifically associated with increased mortality, pulmonary inflammation and oxidative stress [8-10]. Ozone (O_3_) exposure has also been associated with asthma-related hospital visits [11]. Fine particulate matter and O_3 _typically occur together in urban settings [7]. Therefore, it is important to understand the combined effects of these criteria air pollutants on cardiopulmonary disease. In particular, the role of these pollutants in asthma exacerbations remains to be fully understood.

Studies of gene-environment interactions have focused on the role of oxidative stress-responsive genes and air pollution exposures in asthma [12,13]. However, the mechanism(s) linking exposure to air pollution and asthma exacerbation remains unclear. The metabolism of L-arginine plays an important homeostatic role in the airways, through synthesis of the bronchodilating molecule, nitric oxide (NO), from L-arginine, by the nitric oxide synthase (NOS) isozymes [14]. The arginase isozymes (arginases 1 and 2), convert L-arginine into L-ornithine and urea, and thus compete with the NOS isozymes for substrate [15]. We and others have shown that arginase expression is upregulated in human asthma [16-18] and that the arginase isozymes play a functional role in the airways hyperresponsiveness (AHR) in animal models of asthma, using ovalbumin (OVA) [16,17,19,20], *Aspergillus fumigatus *[17], trimellitic anhydride exposure [21], and more recently house dust mite [22]. We have previously demonstrated that the AHR in a chronic murine model of allergic airways inflammation to OVA is due to arginase 1 overexpression [16]. Furthermore, single nucleotide polymorphisms of arginase 1 have been specifically associated with responsiveness to bronchodilators, and L-arginine bioavailability can impact airflow in asthma [23,24].

The arginase pathway has not previously been examined as a potential mechanism underlying the air pollution-induced exacerbation of asthma symptoms. However, arginase has been shown to be further upregulated in smoking asthmatics who are regularly and voluntarily exposed to high levels of particulate matter [25]. Furthermore, there is evidence to support uncoupling of the endothelial NOS in the vasculature following exposure to diesel exhaust [26], and dysfunction of endothelial-dependent vasorelaxation following exposure to second-hand tobacco smoke [27], likely as a consequence of a reduction in the bioavailability of L-arginine or tetrahydrobiopterin for the NOS pathway. Thus, it is plausible that dysregulation of L-arginine metabolism as a consequence of air pollution-induced upregulation of pulmonary arginase could contribute to the exacerbation of respiratory symptoms in susceptible asthmatics. We tested the hypothesis that arginase expression is augmented in response to exposures to environmental air pollutants, using two independent murine models of allergic airways inflammation; sub-acute and chronic models that mimic the inflammatory response and airways remodeling/AHR, respectively [28-31]. We demonstrate further upregulation of arginase following exposure to air pollution and attenuation of the pollution-induced AHR following treatment with an arginase inhibitor in both murine models of allergic airways inflammation.

## Methods

### Sub-acute and chronic models of allergic airways inflammation

**Figure 1:**
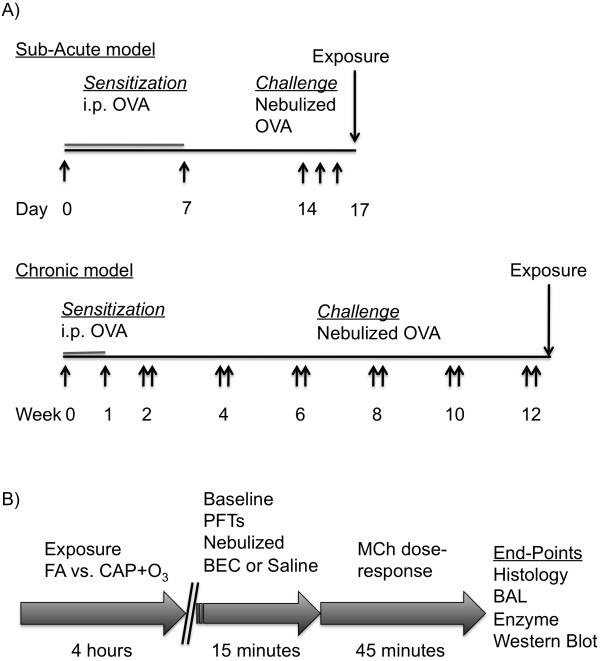
**Experimental design and time-course**. A) Schemas of the sensitization and challenge regimens of the sub-acute and chronic murine models of allergic airways inflammation. B) Experimental design and time-course of the pollution exposure day.

All protocols were approved by the University of Toronto Faculty Advisory Committee on Animal Services, and were conducted in accordance with the guidelines of the Canadian Council on Animal Care, ensuring that the animals were treated humanely. To investigate the role of arginase in the exacerbation of airways responsiveness induced by air pollution exposure, we utilized two murine models of allergic airways inflammation: the sub-acute (16-day) and chronic (12-week) OVA-sensitization and -challenge models, which represent short-term allergic inflammatory changes and remodeling/hyperresponsiveness of the airways, respectively [30,31]. In both models, female BALB/c mice (6-8 weeks of age; Charles River Laboratories, Saint-Constant, PQ) were sensitized to OVA (25 μg i.p. in 0.2 ml PBS with 1 mg Al(OH)_3_; Sigma Aldrich, Mississauga ON) one week apart (days 0 and 7), as described previously [16]. In the sub-acute model, the sensitized mice were randomized into two inhalation challenge groups (nebulized 6% OVA (OVA/OVA) or PBS (OVA/PBS)) for 25 minutes/day from days 14-16 (Figure 1A). In the chronic model, OVA-sensitized mice were challenged with nebulized 2.5% OVA, on two consecutive days followed by a 12-day rest period (i.e., 2-week intervals), for up to 12 weeks (Figure 1A). For both models, 24 hours after the final OVA or PBS challenge, mice were exposed to concentrated ambient particles plus ozone (CAP+O_3_) or HEPA-filtered lab air (FA), as described below, and depicted in Figure 1B.

### Air Pollution Exposures

Combined exposures to CAP and O_3 _were employed in this study. For controlled exposures to concentrated ambient fine particulate matter, we used the Harvard Ambient Particle Concentrator [32], which is a high-flow (5000 L/min) three-stage virtual impactor system that is part of the Southern Ontario Centre for Atmospheric Aerosol Research at the Gage Occupational and Environmental Health Unit. In this system, ambient air is drawn in, and real-world particles with an aerodynamic diameter 0.1-2.5 μm are concentrated approximately 40-fold (range: 196-954 μg/m^3^). O_3 _was produced by an arc generator using medical-grade oxygen and was introduced into the transition plenum between the second and third stages of the concentrator. CAP and O_3 _levels (>175 μg/m^3 ^and 2 ppm, respectively) were selected based upon previous inhalation exposure studies in rodents [33-35]. Mice were exposed to CAP+O_3 _or FA for 4 hours at a flow rate of 2 L/min (Figure 1B) using a modified inExpose nose-only inhalation system (Scireq Inc., Montréal, PQ) within a Plexiglas chamber. The O_3 _levels achieved using this system were monitored on the outflow from the chamber, using a Dasibi Model 1008RS ozone analyzer (Dasibi Environmental Corp, Glendale CA), and particle levels were determined gravimetrically (Table 1). In a subset of exposures, the constituents of the CAP were measured and the levels of major constituents (i.e., organic and elemental carbon, NO_3_^-^, SO_4 _^2-^, and NH_4_^+^) were found to be consistent with our previous analyses of PM_2.5 _in Toronto [36] (data not shown). As our nose-only exposure system allows for the simultaneous exposure of 6 mice, CAP+O_3 _and FA exposures were conducted on 3 OVA/OVA mice and 3 OVA/PBS controls at a time, to ensure comparable exposures between groups. Preliminary experiments indicated that the increase in methacholine responsiveness following exposure to CAP+O_3 _was greater than that to either CAP or O_3 _alone (data not shown).

**Table 1:** CAP and ozone exposure levels for the sub-acute and chronic models

	**CAP (μg/m**^**3**^**) **^**a**^	Ozone (ppm)
Sub-acute	553 ± 79	1.80 ± 0.07

Chronic ^†^	456 ± 44	1.79 ± 0.04

^a ^Values represent the mean ± standard error of n = 8-11 exposures.

^† ^There were no significant differences between the exposure levels for the sub-acute and chronic model mice.

### Pulmonary Function Testing and Arginase Inhibition

Following the CAP+O_3 _or HEPA FA exposures, mice were anesthetized with ketamine (50 mg/kg i.p., Bioniche, Belleville, ON)/xylazine (10 mg/kg i.p., Bayer Inc., Toronto, ON) for measurement of *in vivo *airways responsiveness to methacholine using the flexiVent^® ^system (SciReq Inc., Montréal QC) [16]. The arginase inhibitor, *S*-boronoethyl L-cysteine (BEC; 40 μg/g body weight) or the PBS vehicle were nebulized directly into the airways after establishment of baseline resistance parameters, and allowed to equilibrate for 15 minutes prior to pulmonary function testing, in randomly selected mice from each model. We have previously found this dose to be effective in inhibiting arginase in acute and chronic murine models of asthma [16,20]. Respiratory mechanics were assessed using the linear first-order single compartment model, which provides resistance of the total respiratory system (R), and the constant phase model, which utilizes forced oscillation to differentiate between airways resistance (R_N_) and peripheral tissue damping (G) [30,37,38]. Following pulmonary function testing, bronchoalveolar lavage (BAL) was performed in a subset of mice, for assessment of inflammation and 8-isoprostane as a marker of oxidative stress. All remaining lungs were harvested for protein analysis or immunohistochemical staining.

### Arginase activity and isozyme expression

Total arginase activity testing and Western blotting for arginases 1 and 2 were performed as described previously [16]. Semi-quantitative assessment of the Western blots was conducted using a Bio-Rad Fluor-S MultiImager with the Bio-Rad Quantity One 4.3.0 software package (Bio-Rad Laboratories, Hercules, CA). Densitometry was performed using GelEval v1.22 (FrogDance Software, Dundee UK).

### Inflammation and Assessment of Immunohistochemical Localization of Arginase 1

Differential cell counts were performed on cytospin slides (Shandon, Thermo Scientific, Waltham, MA), stained with DiffQuick (Dade Behring Inc., Newark, NJ). Differential cell counts were performed under a light microscope, by counting more than 300 cells per slide. Immunohistochemical staining of BAL cells and histological sections was performed using standard protocols at the Toronto Centre for Phenogenomics Pathology Core Facility, as previously described [16]. Goat anti-arginase 1 primary (sc-18351) and donkey anti-goat secondary (sc-2042) antibodies were purchased from Santa Cruz Biotechnologies (Santa Cruz, CA). For immunohistochemical counts of arginase 1-positive macrophages, macrophages were identified based on size and morphology using a hematoxylin counterstain. Lungs were collected for immunohistochemical staining and inflated to a pressure of 20 cmH_2_O with 10% neutral buffered formalin (Sigma, Mississauga ON) [39]. For immunohistochemical analyses of tissue arginase 1 expression, slides were visualized on a Leica inverted microscope and images were captured using a micropublisher RTV 5.0 camera with QCapture image capture software (Quorum Technologies Inc., Guelph, ON).

### Oxidative Stress Marker

As a marker of oxidative stress, 8-isoprostane levels (8-*iso*-prostaglandin F_2α_) were measured in BAL fluid using an enzyme immunoassay kit (8-Isoprostane EIA Kit. Item No. 516351, Cayman Chemical Company, Ann Arbor, MI), according to the manufacturer's instructions and standardized to protein concentration in the BAL, as determined by Bradford assay (BioRad, Hercules, CA).

### Statistics

Statistical analyses were performed independently on the data from the sub-acute and chronic models. Specific respiratory measurements (R, R_N_, G), arginase activity and Western blotting densitometry data were analyzed using one-way ANOVA with Bonferroni's multiple comparison post-hoc test. BAL differential cell counts were analyzed using the non-parametric Kruskal-Wallis test with Dunn's Multiple Comparison post-hoc test, as some cell types were not observed in the OVA/PBS controls (i.e., eosinophils). Dose-response curves were compared using the F-test, with the null hypothesis that the data from all groups could be modelled by the same curve, and using two-way ANOVA with Bonferroni's post-hoc test. Correlations between exposure parameters and protein expression were determined by Spearman's test. P-values < 0.05 were considered significant. All statistical analyses were performed using GraphPad Prism 4.0c.

## Results

### Arginase activity and expression

**Figure 2:**
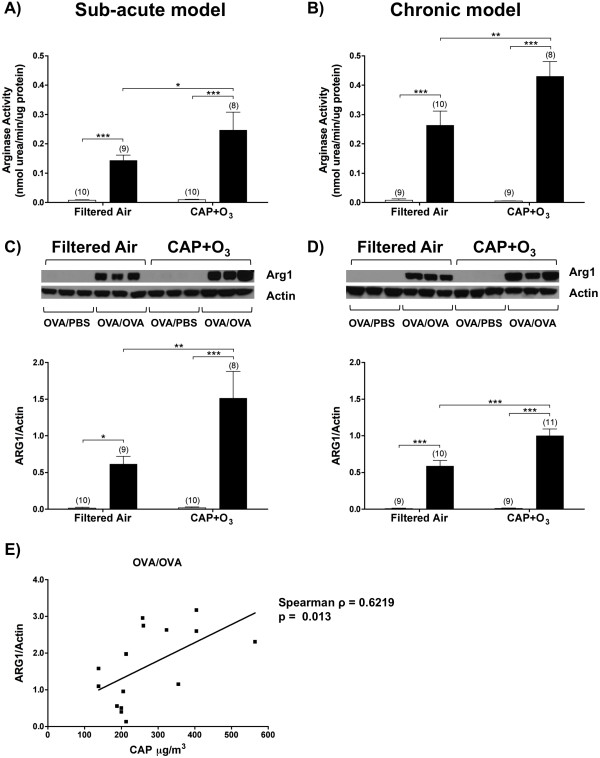
**Pulmonary arginase activity and arginase isozyme expression in CAP+O_3_-exposed mice and filtered air controls**. Total arginase activity in FA- and CAP+O_3_-exposed model OVA/PBS (□) and OVA/OVA (■) mice in the sub-acute (A) and chronic (B) models. Western blotting and quantification of arginase 1 and actin loading controls in the sub-acute (C) and chronic (D) models (*P < 0.05, **P < 0.01, ***P < 0.001, (n)). E) Correlation between levels of arginase 1 expression in the OVA/OVA mice in the sub-acute model and CAP exposure concentration (Spearman ρ = 0.6219; P = 0.013, n = 11 independent exposure dates).

To investigate whether alterations in the arginase pathway were induced by exposure to air pollution we measured total arginase activity in mouse lung homogenates from FA and CAP+O_3 _exposed mice. FA-exposed OVA/OVA mice from both models exhibited significantly increased pulmonary arginase activity, relative to the FA-exposed OVA/PBS controls (Figure 2A &2B). In both models, OVA/OVA mice exposed to CAP+O_3 _exhibited further significant increases in pulmonary arginase activity, compared to the FA-exposed OVA/OVA mice (1.7- and 1.6-fold, respectively). CAP+O_3 _exposure did not affect total pulmonary arginase activity in the OVA/PBS mice.

We used Western blotting to determine the contribution of the arginase isozymes to the increased total arginase activity. Arginase 1 expression was significantly increased in lungs from FA-exposed OVA/OVA mice in both models, relative to their respective OVA/PBS controls (Figure 2C &2D). Following exposure to CAP+O_3_, OVA/OVA mice in the sub-acute and chronic models exhibited further significant increases in pulmonary arginase 1 expression, relative to the FA exposed OVA/OVA controls (2.6- and 1.7-fold, respectively). Interestingly, in the sub-acute model, the pulmonary expression of arginase 1 correlated directly with CAP exposure levels at concentrations lower than 565 μg/m^3 ^(Spearman ρ = 0.622, P = 0.013; linear regression r^2 ^= 0.32; n = 15 mice from 11 independent exposure days) (Figure 2E), suggesting that the CAP-induced increase in expression of arginase 1 was dose-dependent. At exposure levels above 565 μg/m^3 ^we observed no further increase in arginase 1 expression, indicating a plateau in the response at higher levels. As the ozone exposures were fixed at the target concentration of 2 ppm, there was no correlation with protein expression. While pulmonary arginase 2 protein expression was increased significantly in the sub-acute model OVA/OVA mice under FA conditions, it was not further augmented by CAP+O_3 _exposure. No significant increases in arginase 2 protein expression were observed in the chronic model mice, regardless of whether they were exposed to FA or CAP+O_3_.

### Localization of increased arginase 1 expression

**Figure 3:**
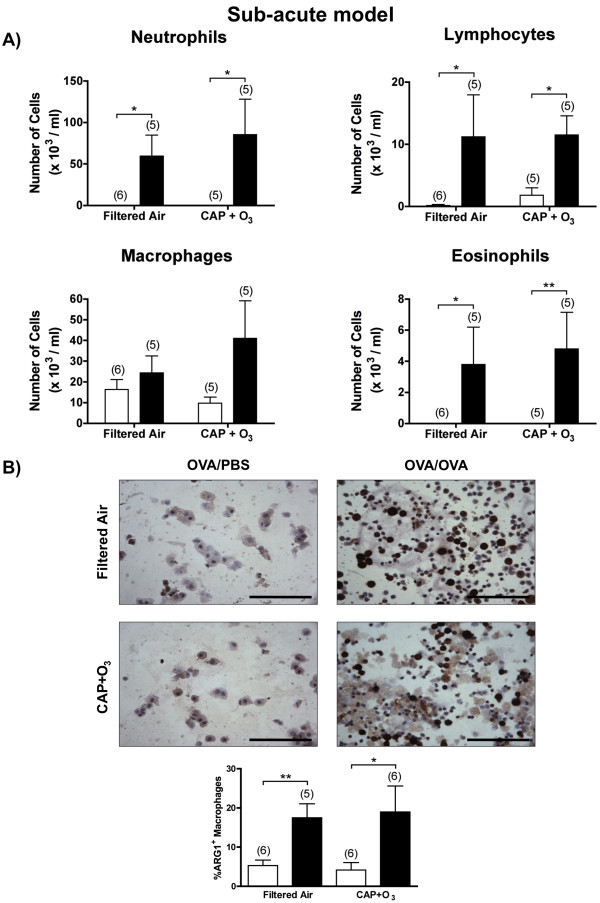
**Bronchoalveolar lavage differential cell counts and macrophage expression of arginase 1**. A) Differential cell counts from BAL samples in the sub-acute model OVA/PBS (□) and OVA/OVA (■) mice exposed to FA or CAP+O_3 _(*P < 0.05). (B) Images of arginase 1 immunostained slides of BAL samples and quantification of the percentage of positive macrophages (400× magnification; bar = 100 μm; brown colour indicates positivity; representative images of n = 5-6/group; *P < 0.05, **P < 0.01).

To determine which cell types were responsible for the augmented arginase 1 expression following exposure to CAP+O_3_, we investigated BAL and lung tissues, using immunohistochemical staining. We first examined the differential cell counts of the BAL samples from the sub-acute model. While there was an overall increase in the numbers of inflammatory cells in the OVA/OVA compared to OVA/PBS mice, there were no significant alterations in the differential cell counts in the CAP+O_3 _compared with the FA exposure groups (Figure 3A).

As arginase 1 is known to be expressed in alternatively-activated macrophages [40], we investigated arginase 1 expression in BAL cells using immunohistochemistry. We did not observe any change in the proportion of arginase 1-positive macrophages in the immunostained BAL slides from the CAP+O_3_-exposed OVA/PBS or OVA/OVA mice compared to their respective FA controls (Figure 3B). Thus, the increase in arginase 1 expression in the CAP+O_3_-exposed mice was not due to an increased proportion of alternatively-activated macrophages infiltrating the lung.

**Figure 4:**
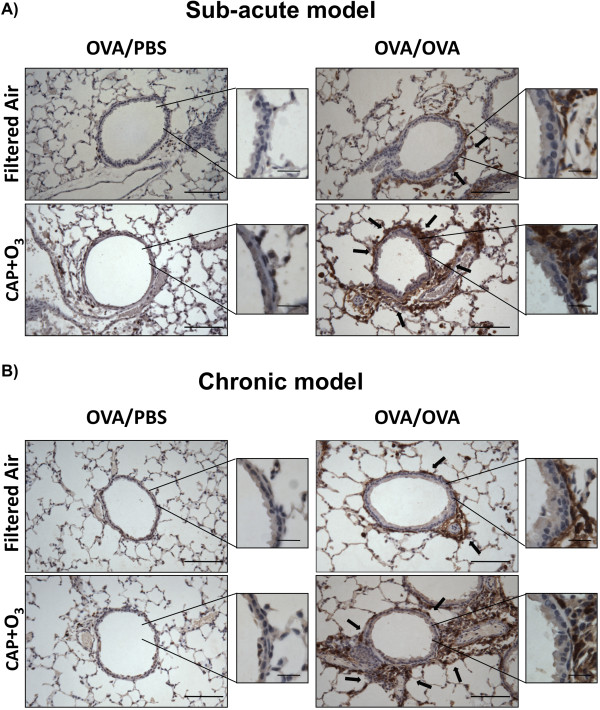
**Immunohistochemistry in CAP+O_3 _and FA exposed mice**. Arginase 1 immunostained lung tissues from OVA/PBS, OVA/OVA mice from the sub-acute (A) and chronic (B) models exposed to filtered air or CAP+O_3 _(200× magnification; bar = 100 μm; representative images of n = 4-5 per group. Brown colour indicates immunopositivity, arrows highlight positive areas, key positive areas inset at 400× magnification; bar = 20 μm).

We then investigated the expression of arginase 1 in airways in lung sections using immunohistochemical staining (Figure 4). Although expression was not quantifiable by these methods, staining was localized to the peribronchiolar region in both the sub-acute (Figure 4A) and chronic (Figure 4B) models.

### Effects of air pollution on methacholine responsiveness

**Figure 5:**
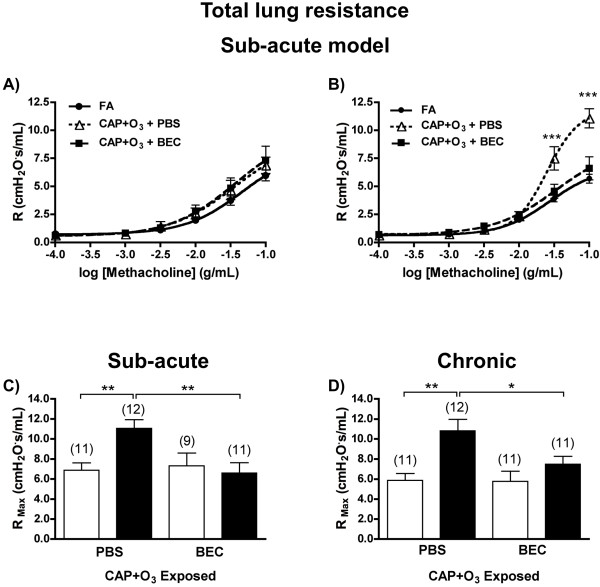
**Functional effects of CAP+O_3 _exposure on airways responsiveness to methacholine and attenuation by arginase inhibition**. Dose-response relationships for the increase in total lung resistance (R) to methacholine in OVA/PBS (A) and OVA/OVA (B) mice from the sub-acute model exposed to FA or CAP+O_3_. Effects of treatment with arginase inhibitor (BEC) *vs*. vehicle control (PBS) on maximum total lung resistance (R_Max_) in OVA/PBS (□) and OVA/OVA (■) mice following CAP+O_3 _exposures in the sub-acute (C) and chronic (D) models (*P < 0.05, ** P < 0.01, *** P < 0.001; n = 9-12/group).

After demonstrating augmentation of arginase 1 protein expression in OVA/OVA mice exposed to CAP+O_3_, we initially examined the functional effects of air pollution exposure on methacholine responsiveness *in vivo *in the sub-acute model. Total lung resistance (R) to methacholine was not significantly augmented in the OVA/OVA mice compared to OVA/PBS controls under FA conditions (Figure 5A and 5B), making this model suitable to investigate the development of AHR induced specifically by CAP+O_3 _exposure. Exposure to CAP+O_3 _did not evoke any significant change in the methacholine responsiveness of the total lung in OVA/PBS mice (Figure 5A). However, significant augmentation of the methacholine dose-response curve was observed in the CAP+O_3_-exposed OVA/OVA mice, with a two-fold increase in the maximum resistance to methacholine, compared with the FA-exposed OVA/OVA controls (F-test and 2-way ANOVA, P < 0.001, Figure 5B and 5C). In the chronic model, FA-exposed OVA/OVA mice exhibited a moderate 1.5-fold increase in methacholine responsiveness compared with the OVA/PBS, FA-exposed controls (P = 0.0418), which was further augmented by 1.6-fold in CAP+O_3_-exposed OVA/OVA mice (P = 0.0071)(Figure 5D).

### Arginase inhibition abrogates the CAP+O_3_-induced AHR

After determining that exposure to CAP+O_3 _resulted in exacerbation of methacholine responsiveness in mice with pre-existing allergic airways inflammation, paralleling the up-regulation of pulmonary arginase 1, we administered the arginase inhibitor, BEC, or vehicle control (PBS) to randomly selected sub-groups of mice following the CAP+O_3 _exposures in both the sub-acute and chronic models. The maximum total respiratory resistance (R_Max_) was significantly increased in OVA/OVA mice vs. OVA/PBS from both models after the CAP+O_3 _exposure (Figure 5C and 5D). After treatment with BEC, the R_Max _values in the CAP+O_3_-exposed OVA/OVA mice was significantly attenuated compared with the PBS-treated controls (i.e., CAP+O_3_-exposed OVA/OVA mice), and were indistinguishable from the R_Max _for the OVA/PBS controls. Thus, treatment with the arginase inhibitor completely reversed the CAP+O_3_-induced exacerbation of symptoms in the OVA/OVA mice.

**Figure 6:**
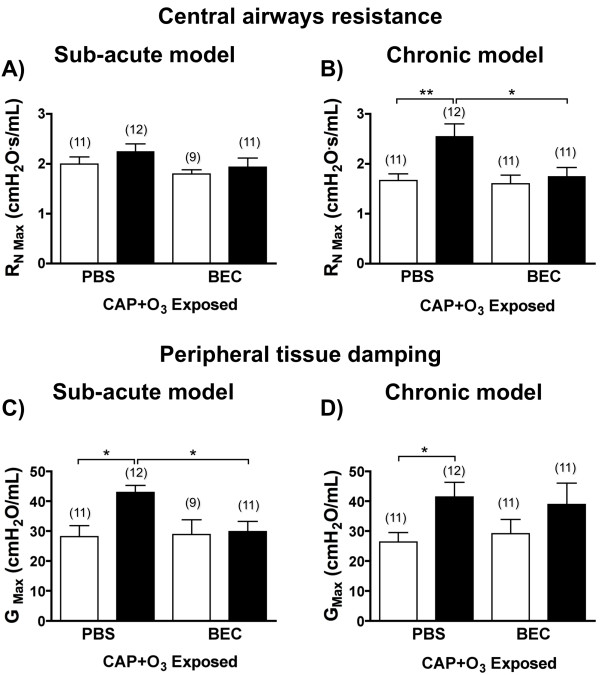
**Arginase inhibition in CAP+O_3 _exposed mice**. Effect of treatment with arginase inhibitor (BEC) *vs*. vehicle control (PBS) on central airways Newtonian resistance (R_NMax_; A and B) and peripheral tissue damping (G_Max_; C and D) in OVA/PBS (□) and OVA/OVA (■) mice from the sub-acute (A and C) and chronic (B and D) models following CAP+O_3 _exposures (*P < 0.05, **P < 0.01, n = 9-12/group).

To confirm that the exacerbation of symptoms was due to effects on the airways, we assessed the contribution of airways resistance (R_N Max_) and peripheral tissue damping (G_Max_) to the total response of the lung. In the sub-acute model, R_N Max _was not altered significantly following CAP+O_3 _exposure, or by BEC treatment (Figure 6A). Interestingly, G_Max _was increased significantly following exposure to CAP+O_3 _in the sub-acute OVA/OVA mice, and was attenuated to control levels by arginase inhibition with BEC (Figure 6C). Meanwhile, in the chronic model OVA/OVA mice, R_N Max _was significantly augmented by CAP+O_3_, and significantly reversed by treatment with BEC (Figure 6B). A significant increase in G_Max _was also observed in the chronic model OVA/OVA mice following CAP+O_3 _exposure, however this was not attenuated by BEC treatment (Figure 6D). Exposure to CAP+O_3 _or administration of BEC did not affect any of the responsiveness parameters in the OVA/PBS mice in either model (Figure 5 and 6).

### Oxidative Stress Due to CAP+O_3 _Exposures

To assess the level of oxidative stress induced by exposure to CAP+O_3_, we determined levels of 8- prostaglandin F_2α _(8-isoprostane) in BAL supernatants from both the sub-acute and chronic models (Table 2). In the sub-acute model, the levels of 8-isoprostane were 7.9 ± 3.6 and 9.7 ± 4.1 pg/mg of BAL protein in the OVA/PBS and OVA/OVA FA groups, respectively (P = n.s.). OVA/PBS and OVA/OVA mice exposed to CAP+O_3 _exhibited 5.4- and 7.0-fold increases compared to the FA groups (P < 0.05 to FA). In the chronic model, BAL levels of 8-isoprostane in the OVA/OVA FA-exposed mice were 1.9-fold greater than those in the OVA/PBS FA-exposed mice (P = 0.017). OVA/PBS and OVA/OVA mice exposed to CAP+O_3 _exhibited 3.5- and 2.3-fold increases in 8-isoprostane levels compared to their respective FA controls (P < 0.05). There was no significant difference in BAL 8-isoprostane levels between the OVA/PBS and OVA/OVA CAP+O_3_-exposed groups.

**Table 2:** 8-isoprostane levels in BAL samples from the sub-acute and chronic OVA-model mice exposed to filtered air or CAP+O_3_.

	**Filtered Air **^**a**^		**CAP+O**_**3**_	
	
	OVA/PBS	OVA/OVA	OVA/PBS	OVA/OVA
Sub-acute	7.9 ± 3.6	9.7 ± 4.1	42.5 ± 11.4^#^	67.4 ± 22.4^#^

Chronic	19.0 ± 3.3	36.8 ± 5.0*	65.7 ± 16.0^#^	83.0 ± 17.0^#^

^a ^Values are expressed as pg 8-isoprostane/mg BAL protein and represent the mean ± standard error of n = 5-6 samples.

* P < 0.05 to OVA/PBS, same exposure; ^# ^P < 0.05 to Filtered air, same treatment.

## Discussion

This study demonstrated that the increased arginase activity in the lungs of mice from both sub-acute and chronic models of allergic airways inflammation was further augmented by exposure to CAP+O_3_, and that this was primarily driven by arginase 1. We also determined that the up-regulation of arginase 1 in the lung was not related to increased influx of macrophages. Finally, we demonstrated that induction of AHR by CAP+O_3 _was specific to the mice with pre-existing allergic airways inflammation, and that local delivery of an arginase inhibitor after exposure, significantly reduced the CAP+O_3_-induced AHR in both models; thus providing further support for the potential of targeting this pathway therapeutically in asthma.

### Arginase induction by CAP+O_3_

There is increasing evidence to support the role of arginase in the pathophysiology of asthma, and that further up-regulation of arginase likely results in worsening of asthma symptoms [15-19]. The sub-acute model mice in the present study, challenged with ovalbumin daily for three days, exhibited significantly lower arginase 1 expression and airways responsiveness, compared to the acute OVA-model mice reported in our previous study, in which we employed seven consecutive daily challenges [16]. Thus, increased arginase 1 expression is directly associated with the increasing airways responsiveness in these murine models (P = 0.002, Spearman ρ = 0.522). We speculate that there is a critical threshold of arginase induction, at which the increased arginase activity exhibits physiological effects. Air pollution is known to contribute to asthma exacerbations [41-43]. Increased levels of particulate matter and ozone have been associated with increased oxidative stress and decreased pulmonary function in children with asthma [44]. Increased arginase protein expression has been observed in smokers with asthma [25], but it is not known whether arginase plays a role in air pollution-induced exacerbations of respiratory symptoms. In this study we demonstrated further augmentation of arginase activity and arginase 1 expression in the airways of our OVA-sensitized and -challenged mice following exposure to CAP+O_3_.

Arginase 1 protein expression in blood serum has recently been associated with markers of oxidative stress in a healthy human population [45], and augmented arginase activity correlates with indices of oxidative stress (i.e., malondialdehyde and protein carbonylation levels) in platelets and plasma from patients with chronic obstructive pulmonary disease exposed to wood smoke [46]. Arginase 1 has also been reported to be induced in isolated coronary arterioles following one hour of *ex vivo *exposure to hydrogen peroxide-induced oxidative stress [47]. Thengchaisri *et al*. also demonstrated changes in smooth muscle function in response to increased oxidative stress, and demonstrated that arginase inhibition restored the hydrogen peroxide-impaired vasodilation [47]. However, in our model we only observed an increase in arginase 1 in mice that were previously sensitized and challenged with ovalbumin; while, we observed increases in 8-isoprostane levels in the BAL samples from all mice exposed to CAP+O_3 _regardless of the presence or absence of airways inflammation. This may be due to differences in experimental procedures, as Thengchaisri *et al*. exposed isolated coronary arterioles to hydrogen peroxide *ex vivo *[47], while we examined the whole lung following *in vivo *acute CAP+O_3 _exposure. Alternatively, toll-like receptors, the hallmark regulators of the innate immune response to bacterial, viral, and parasitic components, have recently been shown to upregulate arginase 1 via an alternative promoter region [48]. It is highly likely that some of these biologic exposures are relevant to our CAP exposures. Further work is necessary to elucidate the mechanisms underlying the upregulation of arginase 1 in response to environmental stimuli in the presence of an inflammatory response.

While we did not investigate the effects of long-term pollution exposure in these models, our findings raise the interesting question of whether continued exposures would result in chronic upregulation of arginase, and lead to remodeling of the airways. Increased airway wall remodeling has been observed by Dai *et al*., following exposure of rat tracheal explants to Ottawa urban air particles [49]. Diesel exhaust particles can also potentiate airways remodeling in a house dust mite murine model of allergic airways inflammation [50] that is known to exhibit augmented arginase expression [22]. Thus, arginase may be induced as part of the host response to cell damage by air pollution, as it is known to be involved in cell growth and wound healing [51]. As the metabolic pathways downstream of arginase are related to cellular proliferation and collagen biosynthesis, it is likely that augmented arginase expression contributes to airways remodeling in asthma [52]. The role of L-arginine metabolism in the effects of chronic air pollution exposure, and the effects of concomitant inhibition of arginase represent future avenues for investigation.

### Functional improvement of airways hyperresponsiveness with arginase inhibition

Although the arginase pathway has been shown to be functionally involved in the development of AHR *in vivo *following allergen challenges with ovalbumin [16,19,20,53] and house dust mite [22], it was not clear whether this pathway would be functionally important in the exacerbation of AHR induced by air pollution. In this investigation we used two mouse models of asthma, which exhibit the inflammatory changes, remodeling and moderate AHR as symptoms of allergic airways disease; while the OVA/OVA mice in the sub-acute model did not exhibit AHR to methacholine, those in the chronic model did exhibit moderate AHR, which was consistent with our previous report [16]. We further demonstrated exacerbation of the AHR in the chronic model and the development of AHR in the sub-acute model after exposure to air pollution. In both murine models we demonstrated an increase in the maximum total lung resistance following air pollution exposure, and that inhibition of arginase, post-exposure, blocked this effect. We also examined the contribution of the airways and peripheral tissue to the net response. Using a sub-acute model of allergic airways inflammation, Tomioka *et al*. previously demonstrated that the effects of allergic inflammation in this model were more pronounced in the lung periphery and thus affected peripheral lung mechanics more strongly than conducting airways mechanics [38]. However, it was not known how the added challenge of a CAP+O_3 _exposure would affect peripheral lung mechanics in the sub-acute model. Our data suggest that CAP+O_3 _exposure specifically aggravated peripheral lung responsiveness to methacholine in the OVA/OVA mice in this sub-acute model. Arginase inhibition with BEC completely abrogated the augmented G_Max_, strongly suggesting a role for arginase in the functional exacerbation of peripheral AHR by air pollution in the sub-acute model.

The particles we employed were derived from the ambient air in Toronto, Ontario, and particles within a specific size range (0.1-2.5 μm) were concentrated, representing real-world particles. We examined the effects of concentrated ambient particles and ozone, as these pollutants have been shown to be associated with increased asthma exacerbations in humans, and because concomitant exposures have been shown to increase respiratory resistance in mice, thus allowing our study to examine the biological mechanisms responsible for these effects [1,2,11,54]. Murine models present several limitations, as no animal model exhibits all of the clinical features of human asthma [30,55-57]. However, our sub-acute model exhibits airways inflammation and our chronic model recapitulates airways remodeling and mild hyperresponsiveness, all of which are important features of human asthma [28,58], and mice in both models exhibited an even greater degree of airways hyperresponsiveness following air pollution exposures. Asthmatics as a group represent a potential susceptible population that would be more significantly affected by air pollution than those who do not have underlying respiratory disease. Our results support this idea, as we did not observe increased airways responsiveness in the control mice, but demonstrated an increase in mice with pre-existing allergic airways inflammation.

While the doses of particulate matter and ozone employed in this study are high, similar doses have been shown to be useful for studying acute mechanisms of air pollution induced AHR in the setting of allergic airways inflammation and healthy controls [33-35]. Exposure to high levels of ozone can induce pulmonary edema and lung injury [35,59]. While OVA/PBS control mice that were exposed concurrent with the OVA/OVA mice in our study did not exhibit any alterations in inflammatory cell counts or profiles in bronchoalveolar lavage samples obtained immediately after CAP+O_3 _exposures, the possibility remains that our findings could be due solely to the high-level exposures. Interestingly, the OVA/PBS mice exposed to CAP+O_3 _exhibited a slight increase in methacholine responsiveness compared with FA. Similarly, the OVA/OVA mice exposed to CAP+O_3 _that were treated with BEC exhibited a similar increase of methacholine responsiveness, suggesting that pathways unrelated to arginase induction, such as edema, could contribute to this non-significant increase. Thus, further investigations will be necessary to determine whether the pathways activated by acute high-level exposures are similar to those activated after chronic exposures to lower levels of particulate matter and ozone.

The chronic model offers the ability to study exacerbation of established disease, in a model that recapitulates more of the features of chronic human asthma, including remodeling, collagen deposition, smooth muscle hypertrophy, and mild AHR [16,28,60-62]. Furthermore, we have previously shown that the chronic model exhibits alterations in the profile of L-arginine-related protein expression that are most similar to those of human asthma [16]. In the chronic model OVA/OVA mice, we found that both central airways resistance and peripheral tissue damping contributed to the pulmonary response to CAP+O_3 _exposure. However only central airways resistance was attenuated by BEC, suggesting that arginase-independent effects, such as lung edema or the accumulation of endogenous NOS inhibitors (i.e., ADMA)[63,64], may also be induced by exposure to air pollution and contribute to AHR. While we observed a correlation between CAP exposures and arginase induction in OVA-sensitized and -challenged mice, future studies should assess the dose-effects of air pollutants and corroborate the findings with additional model allergens.

## Conclusions

Arginase activity and arginase 1 expression are upregulated following environmental exposures in both sub-acute and chronic murine models of allergic asthma. Pollution-induced AHR is attenuated by arginase inhibition in both models. Thus, induction of arginase 1 is likely a key step in the short-term response to air pollution and inhibition may represent a therapeutic target to treat or prevent environmental pollution-induced exacerbations of allergic airways disease.

## Abbreviations

AHR: airways hyperresponsiveness; BAL: bronchoalveolar lavage; BEC: *S*-boronoethyl-L- cysteine; CAP: concentrated ambient particles; EC_50_: half-maximal effective concentration; FA: filtered air; G: peripheral tissue damping; NOS: nitric oxide synthase; O_3_: ozone; OVA: ovalbumin; PBS: phosphate buffered saline; R: total resistance of the respiratory system; R_N_: resistance of the central airways.

## Competing interests

The authors declare that they have no competing interests.

## Authors' contributions

MN carried out the murine studies, molecular biology, immunohistochemical, pulmonary function analyses and drafted the manuscript. HA conducted the analyses of macrophage arginase 1 expression. NK participated in pulmonary function testing and performed the differential cell counts. BU participated in the design of the exposure system and co-ordination of the air pollution exposures. HG participated in the conception and design of the study and critical revision of the manuscript for important intellectual content. FS participated in the conception and design of the study and critical revision of the manuscript for important intellectual content. JS participated in the conception and design of the study, pulmonary function and statistical analyses, drafting of the manuscript and critical revision for important intellectual content. All authors read and approved the final manuscript.
